# Elderberry Lipophilic and Hydrophilic Bioactive Compounds: Characterization and Extract Encapsulation

**DOI:** 10.3390/foods12234233

**Published:** 2023-11-23

**Authors:** Rubén Domínguez-Valencia, Aurora Cittadini, Mirian Pateiro, Paulo E. S. Munekata, José M. Lorenzo

**Affiliations:** 1 Centro Tecnológico de la Carne de Galicia, Avd. Galicia nº 4, Parque Tecnológico de Galicia, San Cibrao das Viñas, 32900 Ourense, Spain; rubendominguez@ceteca.net (R.D.-V.); aurora.cittadini@unavarra.es (A.C.); paulosichetti@ceteca.net (P.E.S.M.); jmlorenzo@ceteca.net (J.M.L.); 2 Instituto de Innovación y Sostenibilidad en la Cadena Agroalimentaria (IS-FOOD), Universidad Pública de Navarra (UPNA), Campus de Arrosadia, 31006 Pamplona, Spain; 3 Área de Tecnoloxía dos Alimentos, Facultade de Ciencias, Universidade de Vigo, 32004 Ourense, Spain

**Keywords:** *Sambucus nigra* L., plant extracts, antioxidants, encapsulation, natural ingredients, functional food

## Abstract

There are few studies on the use of elderberry in the food industry, and its form of application differs between the different studies. Therefore, the objective of this study is to describe a procedure for obtaining a stabilized product with a high content of hydrophilic bioactive compounds (encapsulated elderberry extract). Moreover, the solid residue resulting from the extraction of the polyphenols was characterized, and the lipophilic compounds retained in this residue were analyzed. The results show an important antioxidant activity of the extracts obtained, mainly linked to the high content of anthocyanins, hydroxycinnamic acids, and flavonols. The lipophilic bioactive compounds were characterized by a high content of essential fatty acids and high proportions of tocopherols. The information and results of the present study provide novel information about both lipophilic and hydrophilic compounds for the integral valorization of elderberries to promote a circular economy strategy.

## 1. Introduction

Elderberry is a robust and underutilized fruit used for value-added products [1]. The elderberry crop has shown a great capacity to adapt to many climate conditions [2]. For this reason, there is an increasing interest in wild-growing plant species rich in bioactive compounds [3]. In line with this, high amounts of elderberries from wild-growing shrubs are used for industrial purposes [4]. The black elderberry (*Sambucus nigra* L.) is widely distributed throughout Europe, with the majority of its market localized in Austria, the Czech Republic, Denmark, Germany, Italy, and Poland [5,6], although their production is small in comparison with other berries.

Elderberry is still rarely applied in food formulation, although it has been used in the food industry, mainly for the production of beverages (juices, tea, and alcoholic beverages) [7], as well as coloring in candies, drinks, jam, and confectionaries [8,9]. Elderberry contains one of the highest amounts of anthocyanins in comparison with other berries [10,11,12]; thus, there is an increased interest in this natural source to develop food colorants. It also contains significant amounts of other polyphenols with potential antioxidant activity. In addition to these hydrophilic compounds, other studies have provided convincing evidence that lipophilic substances also have a vital biological impact on human health and positive implications in foods. These substances include essential fatty acids (C18:2 *n*-6 and C18:3 *n*-3) as well as other compounds such as tocopherols. Therefore, the high amount of essential fatty acids, the specific ratio *n*-6/*n*-3, and the important content of tocopherols in the elderberry are also vital for the valorization of this berry [3,13].

However, due to their high water content, they are prone to microbial, chemical, and enzymatic degradation, which makes the marketing of fresh elderberries extremely difficult, and are generally marketed after processing [13]. The heat treatments not only considerably reduce cyanogenic glycosides concentration (up to 96%) in elderberry products [5] but also induce the degradation of elderberry beneficial compounds [14]. Thus, through processing, the stability of the bioactive compounds of elderberries is very low. In fact, various studies have shown that factors such as increased temperature and exposure to light or oxygen dramatically reduce the content of bioactive compounds in elderberry-derived products [15,16].

In order to increase the stability and bioavailability of these compounds, drying and encapsulation procedures have been proposed. To stabilize elderberry products, it is essential to reduce their water activity and moisture [15]. Moreover, several encapsulation techniques, which achieved a double effect (drying and encapsulation), including freeze- and spray-drying procedures, have been developed in recent decades [17,18]. Among all procedures, encapsulation by spray-drying continues to be one of the most widely used [19]. It is characterized by a minimal process duration, simplicity, flexibility, energy efficiency, low operating cost, larger-scale productions, scaling-up technique, high encapsulation efficiency, good stability of encapsulated powder, and available equipment in the market [15,20]. It has been described as an effective method to maintain the stability of anthocyanins throughout storage [21,22] and processing. Another important aspect is the correct selection of an appropriate encapsulant agent to obtain physically stable powders [19]. In this regard, maltodextrin is probably the polymer most used as an encapsulant agent for microencapsulation purposes [18,19]. This carrier produces powders with excellent physical properties and preserves the bioactive compounds [18].

Additionally, the studies published showed several ways to add elderberries to foods (in the form of juice, powder, extract, concentrate, vinegar, etc.); thus, the results and elderberry activity among studies are only somewhat comparable [9]. Consequently, the standardization and complete explanation of extraction and encapsulation conditions described in the present manuscript are vital to producing a reproducible product (encapsulated elderberry extract) to be used in different foods. Furthermore, although there are multiple studies that propose the use of anthocyanin-rich extracts as protective agents for the meat industry due to their antioxidant and colorant properties, such as the use of spray-dried raspberry powder [21], there are no studies on the use of encapsulated elderberry extracts in this industry. In addition, the high concentration of bioactive compounds in the encapsulated elderberry extract also allows food to be enriched with bioactive compounds, thus meeting the growing consumer demand for healthy and functional foods [9,17]. Recent research reported that anthocyanin-rich elderberry extract has a favorable toxicological profile, decreases liver lesions, and exerts a potent defense against oxidative DNA damage [23]. These authors also conclude this natural extract can be used as a food colorant without any negative effects on human health, and might even have some positive effects.

On the other hand, there are multiple studies on the characterization of elderberries, as well as their antioxidant capacity and the optimization of extraction processes [2,3,4,11,18]. However, practically all the studies are focused on phenolic compounds. Nevertheless, there are other important lipophilic bioactive compounds [5,9] that are retained in the residues resulting from the extraction. In addition, dried elderberry pomace has a high content of protein; thus, it could be an alternative vegetal protein source [2]. These residues are mainly composed of peels, pulp residues, and seeds, and they have important compounds that are generally discarded despite their great potential and biological activity [5]. Therefore, in order to promote a circular economy strategy, the integral valorization of elderberries is vital.

Therefore, complete information about the bioactive and valuable compounds present in elderberries is necessary for the rational and integral use of elderberries. The present study proposes not only the characterization of elderberry extracts but also to evaluate the potential of the solid residue resulting from the extraction of phenolic compounds and to fully value all parts of the berries. Currently, there is no study that comprehensively evaluates all potentially extractable bioactive compounds from elderberries, including those retained once the anthocyanins and other polyphenols are extracted. Consequently, the present work has several specific objectives, which include the complete characterization of the hydrophilic compounds extracted from elderberries, the characterization of the lyophilized and encapsulated extracts, the recovery of the resulting solid residue and its chemical analysis, as well as the characterization of the main lipophilic bioactive compounds (fatty acids and tocopherols) retained in the solid residue with the potential to be extracted and used in the food industry. Moreover, the results presented in this manuscript can be used in the development of new and innovative functional ingredients for food applications. In summary, the main objective of the present study was the complete characterization of the main lipophilic and hydrophilic bioactive compounds that can be obtained from elderberries, as well as the encapsulation of hydrophilic compounds.

## 2. Materials and Methods

### 2.1. Invention Information

The technological development and the product (encapsulated elderberry extract) presented in the following sections are protected (ES 1 300 302 U) by a utility model (Application number: U202232132/expedition date: 21 August 2023). The protection includes the process, the final product obtained (encapsulated elderberry extract), and its use as an additive in the meat industry. Therefore, the steps described below are included in the invention protection but do not limit its scope.

### 2.2. Chemicals and Reagents

All reagents were of analytical-grade purity, or HPLC/LC-MS-grade, for chromatographic analysis. 2-Propanol (gradient HPLC grade) (Scharlau; Barcelona, Spain), 2,4,6-tripyridyl-s-triazine (TPTZ) (ThermoScientific Chemicals; Madrid, Spain), 2,2-azinobis-(3-ethyl-benzothiazoline-6-sulphonate) (ThermoScientific Chemicals; Madrid, Spain), 2,20-azobis (2-methylpropionamidine) dihydrochloride (AAPH) (ThermoScientific Chemicals; Madrid, Spain), 2,2-diphenyl-1-picrylhydrazyl (DPPH) (TCL; Paris, France), 37 Component FAME (Supelco; Madrid, Spain), Acetone (VWR Chemicals; Galicia, Spain), acetonitrile (LC-MS grade) (Scharlau; Barcelona, Spain), ammonium sulfate (Scharlau; Barcelona, Spain), boric acid (Carlo Erba Reagents; Barcelona, Spain), chloroform (Scharlau; Barcelona, Spain), DL-α-Tocoferol (Supelco; Madrid, Spain), diethyl ether (Scharlau; Barcelona, Spain), dipotassium phosphate (Scharlau; Barcelona, Spain), ethanol (VWR Chemicals; Galicia, Spain), fluorescein (VWR Chemicals; Galicia, Spain), Folin–Ciocalteu (VWR Chemicals; Galicia, Spain), formic acid (LC-MS grade) (Scharlau; Barcelona, Spain), gallic acid (Scharlau; Barcelona, Spain), hydrochloric acid 37% (Carlo Erba Reagents; Barcelona, Spain), iron (III) chloride hexahydrate (VWR Chemicals; Galicia, Spain), iron (II) sulfate heptahydrate (VWR Chemicals; Galicia, Spain), Kjelcat Cu (Gerhardt; Barcelona, Spain), L(+)-ascorbic acid (Scharlau; Barcelona, Spain), maltodextrin (Ceylan; Valencia, Spain), methanol (Scharlau; Barcelona, Spain), n-Hexane (gradient HPLC grade) (Scharlau; Barcelona, Spain), potassium dihydrogen phosphate (Scharlau; Barcelona, Spain), sodium acetate (VWR Chemicals; Galicia, Spain), sodium carbonate (Scharlau; Barcelona, Spain), Sodium hydroxide (85%) (VWR Chemicals; Galicia, Spain), sodium metal (Acros Organics; Madrid, Spain), sulfuric acid (98%) (Carlo Erba Reagents; Barcelona, Spain), and Trolox (Acros Organics; Madrid, Spain).

### 2.3. Elderberry Material and Experimental Design

**Figure 1:**
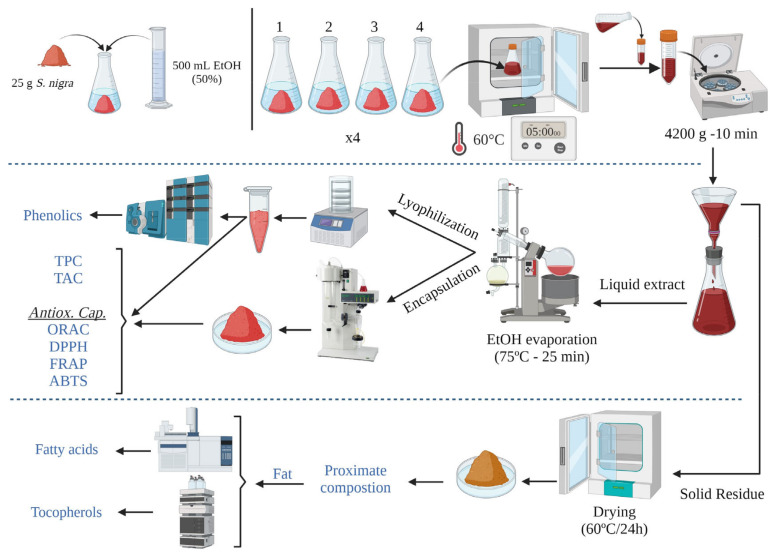
Experimental design for the characterization of bioactive compounds of elderberry.

Wild elderberry samples were collected in quadruplicate (on different days/weeks) at the end of August 2021 in Casas da Veiga (GPS coordinates 42.0716854, −7.8093116) and Trasmiras (GPS coordinates 42.0228502, −7.6176959) (Ourense, Spain). Elderberries (only fully ripe fruits) from each collection (about 500 g of fresh elderberries) were treated as independent samples. They were washed, frozen (−80 °C), lyophilized (Lyovapor L300, Büchi; Barcelona, Spain; primary drying pressure limit: 0.500 mbar, time: 240 min; secondary drying pressure limit: 0.400 mbar, time: 120 min), and ground following the procedure previously described by Domínguez et al. [3]. The lyophilized elderberries (four different samples (approx. 110 g each), depending on the collection) were the material used for the extraction of bioactive compounds, according to the experimental design (Figure 1).

**Figure 2:**
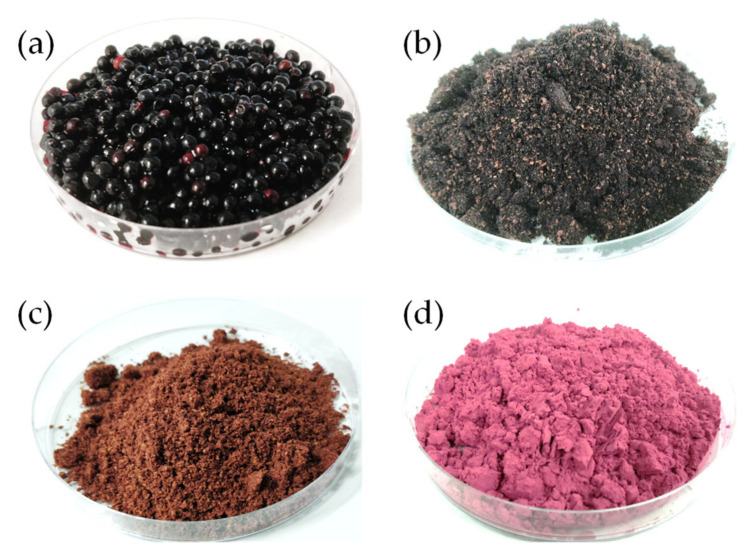
Visual appearance of fresh elderberries (**a**), freeze-dried elderberries (**b**), solid residue after extraction (**c**), and encapsulated extract (**d**).

The extraction parameters and procedure were based on the optimum conditions previously described by Domínguez et al. [3], with modifications. A total of 16 (n = 16) extractions (4 independent extractions per day (for each collection sample) × 4 different days) were carried out. The lyophilized material (25 g) was mixed with 500 mL of ethanolic solution (50%, *v*/*v*) and kept at 60 °C for 5 h (shaking manually every hour). After this time, the samples were centrifuged (4200× *g* for 10 min) and filtered (Filter-lab 1238, Barcelona, Spain) to separate the solid residue from the liquid extract. Ethanol was removed from the liquid extract via rotary evaporation (75 °C for 25 min under vacuum conditions), and the resulting aqueous extract was subdivided into two parts. Half of the extract was freeze-dried following the previously described conditions, while the other half was encapsulated using maltodextrin as the encapsulant agent (13.5 g maltodextrin/100 mL of extract). The encapsulation procedure was carried out in a spray-dryer (Mini Spray-Dryer B-290; Büchi, Barcelona, Spain) with a 145 °C inlet temperature, 28 m^3^/h of aspirator flow (80%), and 600 g/h extract/maltodextrin feed flow [24]. The main groups of bioactive compounds (total phenolic compounds and total anthocyanins) (see Section 2.5.1) and the antioxidant capacity (see Section 2.5.3) were analyzed for both extracts. Additionally, on the lyophilized extracts, the phenolic compounds profile (anthocyanins and non-anthocyanins; see Section 2.5.2) responsible for their antioxidant capacity and bioactive character were analyzed and quantified via liquid chromatography coupled with mass spectrometry. On the other hand, the solid residue obtained after filtration was dried in an oven (60 °C for 24 h) and ground. In order to evaluate the potential of bioactive compounds that are still present in the residue after extraction, the chemical composition (see Section 2.4.1) and the lipid fraction (fatty acids and tocopherols; see Section 2.4.2 and Section 2.4.3, respectively) were analyzed. Figure 2 shows the visual appearance of the elderberries (fresh and lyophilized), the solid residue, and the encapsulated extract obtained during this research.

### 2.4. Solid Residue Characterization

#### 2.4.1. Proximate Composition

The moisture [25], protein [26], and ash [27] content of the solid residue was measured according to ISO procedures, while total fat was quantified following the American Oil Chemists’ Society method after acid hydrolysis [28]. The carbohydrate content was calculated by difference (carbohydrate content = 100—% protein (dry matter)—% fat (dry matter)—ash (dry matter).

#### 2.4.2. Fatty Acid Composition

The oil extraction was carried out using solvents (methanol and chloroform) under the procedure and proportions described by Barros et al. [29]. After that, 20 mg of oil was transesterified with sodium methoxide and sulfuric-methanol solutions, extracted with n-hexane, and fatty acids were separated, identified, and quantified via gas chromatography according to the method and chromatographic conditions previously described [29]. The results were expressed as g/100 g of oil.

#### 2.4.3. Tocopherol Composition

For the quantification of tocopherols, 100 mg of oil extracted, as indicated in the previous section, was re-dissolved in 3 mL of n-hexane, filtered through nylon syringe filters (0.45 μm), and injected into a liquid chromatograph (HPLC). For the analysis, 10 µL of each sample and standards (α-, β-, δ- and γ-tocopherol) were injected in isocratic mode (2-propanol/n-hexane, 2 and 98 *v*/*v*) at 1 mL/min flow in an HPLC system equipped with Alliance 2695 and 2475 fluorescence detector (Waters, Milford, CT, USA). The tocopherol separation was carried out in a column SunFire™ Prep Silica (4.6 mm ID × 250 mm, 5 μm particle size, Waters, Milford, MA, USA) at 35 °C, and was detected with a fluorescence detector (290 nm λ excitation and 330 nm λ emission). Results were expressed as µg/g oil.

### 2.5. Encapsulated and/or Lyophilized Extract Characterization

#### 2.5.1. Determination of Total Phenolic Compounds (TPC) and Anthocyanins (TAC)

The determination of the main groups of bioactive compounds (TPC and TAC) was carried out using spectrophotometric techniques according to the procedures described by Domínguez et al. [3].

#### 2.5.2. Characterization of Phenolic Compounds

The phenolic profile was determined in the lyophilized extracts re-dissolved in a methanol:water (80:20, *v*/*v*) solution and filtered through a 0.22 µm disposable filter. The chromatographic analysis was performed by LC-DAD-ESI/MS^n^ (Dionex Ultimate 3000 UPLC, Thermo Scientific, San Jose, CA, USA) using the conditions previously described by Bessada et al. [30]. Briefly, the compounds were separated with a reversed-phase column Spherisorb S3 ODS-2C18 (3 µm particle size, 4.6 mm I.D., 15 mm length). The mobile phase (A) was 0.1% formic acid in miliQ water, and phase (B) was acetonitrile. The elution was carried out at 0.5 mL/min flow and in gradient mode: isocratic 15% (B) (5 min); 15–20% (B) (5 min); 20–25% (B) (10 min); 25–35% (B) (10 min); 35–50% (B) (10 min); re-equilibration. Double online detection at 280 and 370 nm wavelengths were used (DAD detector). MS detection was performed in negative mode using a Linear Ion Trap LTQ XL mass spectrometer (Thermo Finnigan, San Jose, CA, USA) equipped with an ESI source. Sheat gas (N_2_) was set at 50 psi; the spray voltage was 5 kV; source temperature was 325 °C; capillary voltage was −20 V; collision energy was 35. The full scan covered the mass range from 100 to 1500 *m*/*z*. Data acquisition was carried out with Xcalibur^®^ data system (ThermoFinnigan, San Jose, CA, USA). The identification of the phenolic compounds was performed using standard compounds, when available, via comparison of their retention times, UV-vis, and mass spectra, and comparing the obtained information with available data reported in the literature, giving a tentative identification. For quantitative analysis, a calibration curve for each available phenolic standard was constructed based on the UV signal. For the identified phenolic compounds for which a commercial standard was not available, the quantification was performed through the calibration curve of the most similar available standard. The results were expressed as mg/100 g of lyophilized extract.

#### 2.5.3. Determination of In Vitro Antioxidant Capacity

Four different techniques were used for the determination of in vitro antioxidant capacity, including oxygen radical absorbance capacity (ORAC), 2,2-diphenyl-1-picrylhydrazyl radical-scavenging activity (DPPH), ferric-reducing antioxidant power assay (FRAP), and 2,2-azinobis-(3-ethyl-benzothiazoline-6-sulphonate) radical (ABTS) according to the procedures described by Franco et al. [31].

### 2.6. Statistical Analysis

A total of 48 samples were analyzed, distributed into 16 samples of each type (lyophilized extract, encapsulated extract and solid residue) (n = 16 each) (4 independent extractions/day × 4 different extraction days). Experimental data were reported as mean values and standard deviations. Analysis of variance (one-way-ANOVA) for comparison of encapsulated and lyophilized extracts was performed using SPSS software (version 25). Differences were considered significant when *p* < 0.05. Pearson’s correlation test was used to verify the correlations between the main groups of bioactive compounds, individual compounds, and the antioxidant capacity of the extracts.

## 3. Results and Discussion

### 3.1. Proximate Composition and Lipophilic Bioactive Compounds of the Solid Residue

After the extraction of the hydrophilic bioactive compounds (mainly anthocyanins and other polyphenols), it is evident that a large mass of material remains in the form of residue. In the present research, the dry solid residue after the extraction of the phenolic compounds represented 46.82 ± 4.96% of the initial mass of lyophilized elderberry. Therefore, it can be affirmed that this residue represents almost half of the initial mass, so there is enormous potential for the extraction of bioactive compounds that are still present in it, and thus achieve the integral use of the elderberry. For this, it is essential to know what types of compounds are present [13], so it was decided to study their chemical composition (Table 1).

Carbohydrates were the main macronutrient of the solid residue (65.91 g/100 g dry matter). As indicated, this solid residue is formed by the insoluble elderberry parts during extraction, so its composition is mainly skin, seeds, and exhausted pulp. With this in mind, it is reasonable that carbohydrates are the major components of this residue. In this case, sugars are soluble in the hydroalcoholic solution used during extraction; thus, the carbohydrates of solid residue are mainly composed of fiber. The same trend was observed by Costa et al. [2], who found that carbohydrates are the main macronutrient (82.4 g/100 g of dry matter) in elderberry dry pomace, while fiber represented 22.4 g/100 g of dry matter. Also, Domínguez et al. [3] observed that the total carbohydrates in elderberries were 79.42 g/100 g dry matter. The higher content of carbohydrates in these studies than in the present work is due to the fact that these authors used complete elderberry or elderberry pomace, which means that they have a high sugar content, while in our case, these simple sugars were removed in the extraction phase.

Another important macronutrient is protein, and solid residue contained important proportions (15.66 g/100 g of dry matter). This value was very similar to those reported by Domínguez et al. [3], who observed 14.08 g protein/100 g of dry matter in elderberries. In contrast, lower protein amounts were found in pomace powder (5.9 g/100 g of dry matter) [2]. Other studies also found lower protein values (2.7–2.9%) in elderberries [5,32]. A possible explanation for this high proportion of protein in our study may be that the removal of important compounds (all hydrophilic compounds) during the extraction process implies that the compounds that are not extracted and remain in the solid residue are concentrated in percentage terms. In addition, it is important to highlight that elderberry protein has a good nutritional value since it contains significant amounts of essential amino acids. In fact, a study reported isoleucine, lysine, and methionine as the limiting amino acids, but elderberry protein may still be considered as a satisfactory source for these amino acids [33]. The other essential amino acids’ chemical scores ranged from 0.7 to 1.4, except for tyrosine (2.03). Moreover, these authors reported a chemical score of 0.94 for total essential amino acids, which indicates that elderberries are a food source of high biological value protein. Thus, taking into account the high proportion of protein in the solid residue, it could be an alternative vegetal high-quality protein source.

In elderberries, lipids are located primarily in the seeds [5]. Thus, it is expected that solid residue contains important non-polar constituents. In our research, total lipids in solid residue were the second most important macronutrient, and represented 17.07 g/100 g of dry matter. Therefore, an important proportion of lipophilic bioactive compounds could be extracted from this fraction. Our value was similar to those reported in elderberry seeds (10.5 g/100 g of dry matter) by Fazio et al. [13]. In contrast, the lipid content of elderberry pomace (2.5 g/100 g of dry matter) [2] and elderberries (1.6 g/100 g of dry matter) [3] was lower than those observed by us.

In addition to the high oil content observed in the present study, the elderberry fatty acids profile was characterized by a high amount of polyunsaturated fatty acids (PUFA; 65.75 g/100 g of oil), followed by monounsaturated fatty acids (MUFA; 11.8 g/100 g of oil) and very low proportions of saturated fatty acids (SFA; 8.50 g/100 g of oil). Fatty acids represent 86.05 g/100 g of total lipids (Table 1). The major individual fatty acids were C18:2 *n*-6 and C18:3 *n*-3 (about 32 g/100 g of oil each), with important amounts of C18:1 *n*-9 (10.72 g/100 g of oil). This profile was reported by other authors, who observe linoleic and α-linolenic fatty acids as the most important, and significant contents of oleic acid [3,5,13]. In fact, it has been concluded that elderberry oil is an adequate source of healthy fatty acids for a balanced diet [9]. The practically coincident value of the content of C18:2 *n*-6 and C18:3 *n*-3 affects the fact that this oil presents a *n*-6/*n*-3 ratio of 1.01. Following the recommendations of international health institutions, a healthy diet must present a high PUFA proportion, and these must be balanced. The recommended *n*-6/*n*-3 ratio is 4 or less, while the optimal value is 1. In this sense, it is clear that elderberry oil is not only within the recommended value but also has an optimal value of this ratio and is a balanced source of essential PUFA, which agrees with the findings observed in previous studies [3,5,13,32].

According to several epidemiological studies, the intake of PUFA, especially the essential PUFA, like C18:2 *n*-6 and C18:3 *n*-3, contributed to the reduced prevalence of chronic diseases such as cardiovascular diseases, hypercholesterolemia, inflammatory and autoimmune diseases, cancer, and diabetes [5,34]. Thus, both the high amounts of these two essential fatty acids (76% of total fatty acids) and the exceptional *n*-6/*n*-3 ratio indicate that elderberry oil is of high quality, and the oil extraction from solid residue is an opportunity to obtain valuable compounds to produce enriched and functional foods with added value to human health protection.

Regarding other lipophilic bioactive compounds, several antioxidant compounds belonging to tocopherols are present in elderberry solid residue. In our case, the total tocopherol content was 791 µg/g of oil, where the most important tocopherols were δ-tocopherol (463 µg/g of oil) and α- tocopherol (317 µg/g of oil), while we detected minor contents of β- and γ-tocopherol (Table 1). As occurs in the present research, some authors also reported abundant contents of α-tocopherol and γ-tocopherol in elderberry [13,22]. In contrast to our findings, Fazio et al. [13] did not detect β- and δ-tocopherol in elderberry seed oil, while the content of α-tocopherol (0.49 µg/g of oil) and γ-tocopherol (2.63 µg/g of oil) were significantly lower than those observed by us. As occurs with other bioactive compounds, there are many factors that can influence their content, and this can partially explain the differences between the two studies. In addition, Fazio et al. [13] indicated that the seeds were subjected to several stages to obtain the seed flour, including a phase of drying at high temperatures (110 °C for 2 h), which could also explain why these authors have a lower tocopherol content (highly susceptible to heat degradation) than those observed in the present study.

From the nutritional point of view, it is well known that α-tocopherol has the highest vitamin E bioactivity [13]; thus, the elderberry oil extracted from solid residue could be an important source of this vitamin. Additionally, all tocopherols show important antioxidant ability because they transfer hydrogen atoms to radicals, delay hydroperoxide decomposition, and scavenge singlet oxygen [9]. Thus, elderberry oil is also a source of strong antioxidants.

In conclusion, the solid residue, which represents a great proportion (approx. 47%) of initial elderberry mass, contained significant amounts of important macronutrients (lipids and proteins) and also high contents of lipophilic antioxidants and active compounds, such as essential fatty acids and tocopherols. Therefore, the results obtained in this study show the possibility of using the residue after extracting the hydrophilic compounds to obtain important substances while achieving integral use of elderberries, valuing the by-products that are generated during their processing, which are otherwise generally managed as waste and used as animal feed or discarded.

### 3.2. Main Bioactive Compounds and Antioxidant Capacity of Lyophilized and Encapsulated Elderberry Extracts

The extraction conditions of the phenolic compounds significantly impact their activity (antioxidant, colorant, and antimicrobial) as well as the recovery yields [22]. In the present study, the conditions applied for the extraction of phenolic compounds have been based on previous research [3] in which our research group has optimized these conditions to obtain high yields of extracts with excellent antioxidant properties. As specified in the experimental design (Figure 1), part of each hydroalcoholic extract, after removing ethanol content, was directly lyophilized (without carrier), while another part was microencapsulated (spray-drying) using maltodextrin. In this last case, the ratio of the encapsulant agent to the solids of the extract was 2.72 g of maltodextrin/g solids, and the content of solids in the liquid extract after ethanol removal was 4.27 ± 0.19 g solids/100 mL of extract. The encapsulated product yield (mass of powder recovered, considering total solids in the feed solution) obtained by the spray-drying procedure was 69.2 ± 3.07%. This value was lower than those obtained by Murugesan and Orsat [15] using 5:1 ratio proportions (80.1%) and similar to a 1:1 ratio (72.8%) but higher than those reported by Ribeiro et al. [20], who described that the product yield ranged from 25 to 41%. The differences could be attributed to the variations in encapsulant agent proportions and spray-drying conditions.

It is important to highlight that the lyophilized extract presented exceptional hygroscopicity, which makes its handling and storage difficult, limiting its potential use at an industrial level. In contrast, the encapsulated extract remained unaltered and was easily manipulated. Moreover, dehydration allows for obtaining powders rich in bioactive compounds due to the concentration of these compounds [17]. Additionally, reducing water activity is vital for preserving bioactive compounds during storage since dramatic degradative consequences in anthocyanin content were reported as increasing the water activity [10]. In the present study, the main groups of bioactive compounds (total phenolic and anthocyanin contents) and the antioxidant capacity were measured in each extract (lyophilized and encapsulated). These data are presented in Table 2. It is important to note that total phenolic compounds (TPC) assay could be easily affected by other interfering substances, such as sugars, proteins, or ascorbic acid, and chromatographic determinations report more reliable results, but TPC and total anthocyanin content (TAC) can be useful for making general comparisons with other studies [1].

The TPC value in the lyophilized extract was 7486 mg of GAE/100 g, while in the encapsulated extract, this value decreased and was 1132 mg of GAE/100 g. Similarly, TAC also decreases from 520 mg/100 g in the lyophilized extract to 60 mg/100 g in the encapsulated extract. Our TPC and TAC values in the lyophilized extract were higher than those reported in elderberry freeze-dried powders (1242 mg of GAE/100 and 171.8 mg/100 g for TPC and TAC, respectively) by Busso Casati et al. [17], but in our spray-dried powders, these values were similar to those obtained in the other study. These authors use a similar encapsulant/solid ratio (2.12) than in the present research, and both encapsulated powders presented similar results. In contrast, the use of a lower encapsulant/solid ratio (0.41) in elderberry freeze-dried powders resulted in higher total monomeric anthocyanin contents (between 1059 and 1363 mg/100 g) [10]. The present results in lyophilized extracts were higher than those described in our previous study [3], which obtained 2857 mg of GAE/100 g for TPC and 473 mg/100 g for TAC, and similar to those reported in ethanolic extracts obtained by Duymuş et al. [35] (7594 mg of GAE/100 g for TPC and 1066 mg/100 g for TAC). Moreover, in recent research, the authors reported similar values of TPC (1277 mg of GAE/100 g dry matter) in frozen elderberries and higher TAC content (4061 mg/100 g dry matter) [19] than in the present research, while Mattson et al. [18] found 2791 mg of GAE/100 g dry matter for TPC and 1514 mg/100 g dry matter for TAC in elderberry fruit. However, when these authors encapsulated (spray-drying) this extract using maltodextrin as an encapsulant agent (ratio of maltodextrin/solids of 11.2), these values decreased (457 mg of GAE/100 g and 239 mg/100 g for TPC and TAC, respectively). Finally, other authors reported lower TPC values in wild fruit elderberry extract (5678 mg of GAE/100 g dry matter) [4] and elderberries (4802 mg of GAE/100 g dry matter) [36] and higher TAC content (3071 mg/100 g dry matter [4]; 3278 mg/100 g dry matter [36]) than those obtained by us in the lyophilized extract.

In our research, after encapsulation, the TPC was 6.61 times lower, while the TAC was 8.67 times lower than that of the lyophilized extract. This fact agrees with those reported by other authors, who described that both TPC and TAC were considerably lower in spray-dried raspberry powder than in freeze-dried powders [21]. The differences between extracts could be attributed to the presence of an encapsulant agent in the spray-dried powders, while the lyophilized extract did not have any carrier, which creates a “dilution” effect in the spray-dried powders [21]. That is, for the same amount of extract, the lyophilized extract only contains the elderberry, while the encapsulated one has extract and maltodextrin, which determines that the proportions of TPC and TAC are lower since these compounds are only present in the elderberry. The use of encapsulant agents is vital to obtain high-quality powders, but the proportion between encapsulant and solids determines the final characteristics of the powders [10]. In fact, Baeza et al. [10] proposed the use of low encapsulant proportions to obtain elderberry powders with high amounts of anthocyanins and concluded that encapsulated powders with higher anthocyanins content were those obtained using the lowest encapsulant proportion. In any case, as aforementioned, in the present study, the proportion of maltodextrin with respect to solids is 2.72, so it also seems that the drying method, as well as the high temperature applied in the spray-drying, can cause changes (degradation) in the content of these bioactive compounds [15]. This explanation was also supported by Busso Casati et al. [17], who reported that the freeze-drying process produces better conservation of heat-sensitive bioactive compounds and antioxidant capacity than spray-drying.

According to the antioxidant and antiradical activities, the results show a similar trend to that of the TPC and TAC. The values of DPPH decreased from 39.92 mg of Trolox/g in the lyophilized extract to 3.84 mg of Trolox/g in spray-dried powders. ABTS and FRAP values also decreased from 145.9 mg of ascorbic acid/g and 177.7 mmol of Fe^2+^/100 g, respectively, to about 12 in both cases, while ORAC decreased from 286 mg of Trolox/g in lyophilized powders to 46.0 mg of Trolox/g in encapsulated powders. Similarly, the values of IC_50_, which represents the concentration required to scavenge 50% of the free radicals, increased from 4.24 to 34.5 mg/mL when using an encapsulant agent. A lower IC_50_ value refers to a higher antioxidant activity [13]. Therefore, the antioxidant capacity in the encapsulated powders was between 6.2 and 14.5 times lower than the lyophilized powders, depending on the determination method (DPPH: 10.4 times; ABTS: 11.3 times; FRAP: 14.5 times; ORAC: 6.2 times). As has been discussed, the decrease is due to both the dilution effect exerted by the encapsulant agent and possible degradation during the encapsulation process via spray-drying. In a previous study [3], the analysis revealed higher antioxidant activities for DPPH and ABTS but lower for ORAC and FRAP procedures than those reported in the lyophilized extract of this research. It is important to highlight that in the previous study, the results are expressed in dry elderberry, while in this study, the results are expressed in extract, so the differences between both studies could be partially related to this fact. Moreover, there are several other parameters (edaphic, climatic, cultivar, etc.) that can cause variation in the main bioactive compound content and, thus, the antioxidant activity of elderberries. Additionally, in a recent study, the authors demonstrated that wild-growing plants have lower antioxidant capacity than cultivated plants [4], which can also be related to the variations among different studies.

In our case, the antioxidant and antiradical activities were strongly, positively, and significantly correlated with TPC (r = 0.993 (*p* < 0.001) DPPH; r = 0.989 (*p* < 0.001) ABTS; r = 0.990 (*p* < 0.001) FRAP; r = 0.973 (*p* < 0.001) ORAC) and also with TAC (r = 0.994 (*p* < 0.001) DPPH; r = 0.997 (*p* < 0.001) ABTS; r = 0.996 (*p* < 0.001) FRAP; r = 0.990 (*p* < 0.001) ORAC). This fact was also observed in other studies [4,11,13,17,21,35], where positive and strong correlations between phenol and anthocyanin contents and antioxidant and radical-scavenging capacity were described. This high correlation is to be expected since phenols and anthocyanins are strong antioxidants and act as metal chelators, quenching single oxygen and limiting initiation and propagation reactions by scavenging reactive substances [35]. Additionally, the combination of phenolics and anthocyanins exerts a synergistic effect, resulting in an increase in antioxidant capacity.

In general conclusion, encapsulation has led to a significant reduction in the TPC and TAC content, as well as the antioxidant capacity of the extract (mainly due to the dilution effect exerted by the wall material used in encapsulation). However, the encapsulated extract presents significant benefits since the material is much more manipulable and more stable than the lyophilized extract, which would be practically impossible for industry to use. Although it has not been raised in this study, it would be necessary to expand the information on the stability of the encapsulated elderberry extract.

### 3.3. Phenolic Compounds of Lyophilized Elderberry Extracts

The lyophilized elderberry extracts analyzed using the LC-DAD-ESI/MS^n^ technique showed the presence of seven important polyphenolics, which include three non-anthocyanin and four anthocyanin compounds (Table 3). In the non-anthocyanin group, peaks at 5.83 and 6.63 min from the molecular [M-H]^−^ ion at *m*/*z* 353 yielded deprotonated quinic acid (*m*/*z* at 191) as the base peak, and supplementary ions at *m*/*z* 179 (caffeic acid moiety) and at *m*/*z* 173 [37] were identified as “*cis*” and “*trans*” 5-*O*-caffeoylquinic acid, respectively. A peak detected at 16.52 min, with a parent ion [M-H]^−^ ion at *m/z* 609 and loss of quercetin at *m/z* 301, was identified as quercetin-3-*O*-rutinoside (rutin) [37,38]. Additionally, the maximum absorption wavelengths for these compounds agree with those described by other authors [16,36,37,38], which confirms their identification.

With this in mind, our results showed that 5-*O*-caffeoylquinic acids were the most representative hydroxycinnamic acids, while quercetin-3-*O*-rutinoside was the characteristic flavonol in elderberries. The amount of each compound in the non-anthocyanin group was very similar (between 218 and 286 mg/100 g of extract). Our findings agree with several authors [11,16,38,39], who conclude that quercetin-*O*-rutinoside and 5-caffeoylquinic acids are the major non-anthocyanin phenolic compounds in elderberry fruits. Important contents of 5-*O*-caffeoylquinic acid were also reported by other authors in several elderberry fruit cultivars [11,16,38,39].

On the other hand, four different anthocyanins have been detected in the lyophilized elderberry extracts. In this case, the characteristic fragment at *m*/*z* 287 corresponds to the cyanidin aglycone [13]; thus, it is expected that all anthocyanins found in our extracts are cyanidin-derivative anthocyanins. The peak detected at 27.89 min had an ion at [H]^+^ at *m*/*z* 743, which yielded MS^2^ fragments at *m*/*z* 581, 449, and 287. This characteristic fragmentation corresponds to cyanidin-3-*O*-sambubioside-5-*O*-glucoside [12,35].

The peak at 30.97 min was fragmented from a parent ion [H]^+^ at *m*/*z* 581 to 287, indicating a loss of a sambubiosyl moiety. The peak was therefore identified as cyanidin-3-*O*-sambubioside. Moreover, the peak at 31.92 min, with a molecular ion [H]^+^ at *m*/*z* 449 and a major fragment at *m*/*z* 287, was identified as cyanidin-3-*O*-glucoside [12,35,37].

In addition to the characteristic mass fragments, cyanidin-derived anthocyanins showed maximum absorption at the wavelengths of 515–518 nm, which agrees with the wavelengths reported in the literature for these compounds (between 515 and 525 nm) [16,18,35,36,37].

Our results indicated that the cyanidin-based anthocyanins were the major phenolic compounds detected in lyophilized elderberry extracts, which agrees with data reported by several authors [9,12]. Additionally, in accordance with the results of Mattson et al. [18], the only group of anthocyanins in elderberry were cyanidin-based anthocyanins. Among them, the highest contents were observed for cyanidin-3-*O*-sambubioside and cyanidin-3-*O*-glucoside, with very close values (about 4900 mg/100 g of extract each). In agreement with our findings, several authors reported both cyanidin-3-*O*-sambubioside and cyanidin-3-*O*-glucoside as the two major anthocyanins in elderberry [2,10,11,14,16,17,18,23,39]. In the present study, both compounds represented 88% of the total anthocyanins (44.5% for cyanidin-3-*O*-glucoside and 43.6% for cyanidin-3-*O*-sambubioside), and this value was within the range proposed by Domínguez et al. [9], Młynarczyk et al. [6], and Senica et al. [39], who reported that both anthocyanins contribute together around 85–90% of total anthocyanins present in elderberries. Similarly, Busso Casati et al. [17] reported that cyanidin-3-*O*-glucoside was the major anthocyanin in elderberry freeze-dried powders and that it represents 42% of total anthocyanins. Moreover, important amounts of cyanidin-3-*O*-sambubioside-5-*O*-glucoside were also reported by other studies in elderberries [2,11,16,39,40].

Similar to the results reported by Duymuş et al. [35], in the present study, significant correlations were observed between the content of cyanidin-3-*O*-glucoside and the antioxidant activity measured with the FRAP procedure (r = 0.642; *p* = 0.013). Moreover, the content of *trans*-5-*O*-caffeoylquinic acid (r = 0.753; *p* < 0.001) and total non-anthocyanin compounds (r = 0.568; *p* = 0.022) were strongly correlated with the ORAC values, while quercetin-3-*O*-rutinoside content was correlated with FRAP values (r = 0.563; *p* = 0.023). In view of the results, it seems evident that several compounds, including hydroxycinnamic acids, flavonols, and anthocyanins, determine the antioxidant and colorant properties of elderberry extracts.

## 4. Conclusions

In the present study, the effect of encapsulation on the concentration of the hydrophilic bioactive compounds and their antioxidant capacity was investigated. Encapsulant agents exert a dilution effect, which causes a decrease in their antioxidant capacity. However, it should be noted that the encapsulated elderberry powder is more manageable and stable than the lyophilized extract. Additionally, the complete description encapsulant process allows homogenizing the way of obtaining a functional and novel elderberry-derived product that is not present on the market.

The polyphenol profile indicated that the most important hydrophilic compounds in elderberry extract were cyanidin-derived anthocyanins, characterized by high amounts of cyanidin-3-*O*-glucoside and cyanidin-3-*O*-sambubioside. Moreover, significant amounts of non-anthocyanins compounds, such as hydroxycinnamic acids (5-*O*-caffeoylquinic acids) and flavonols (rutin), were also detected. All these compounds determined the high antioxidant properties of elderberry extract.

On the other hand, the solid residue resulting from the extraction of the hydrophilic bioactive compounds represents an important part of the initial mass of elderberry (over 47%). It has high amounts of important macronutrients (oil and protein). Among lipophilic compounds, oil extracted from solid residue has an exceptional fatty acid profile, characterized by its significant amount of PUFA and essential fatty acids (C18:2 *n*-6 and C18:3 *n*-3) and optimal ratio *n*-6/*n*-3, which indicate that this oil is a potential source for the obtention of important lipophilic substances and their use for the enrichment or manufacture of functional foods. Taking into account the increase in demand for natural food ingredients, elderberry may be an additional source of lipophilic and hydrophilic bioactive compounds.

Therefore, as a general conclusion, the entire process of obtaining a novel, stable, and versatile food ingredient (encapsulated elderberry extract) was completely explained and characterized. The strategy applied in this study provides important information about the use of elderberry as a potential source for the obtention of valuable compounds for the food industry. This strategy includes the extraction and stabilization (encapsulation) of hydrophilic compounds (mainly phenolics and anthocyanins), the integral use of solid residue for the extraction of lipophilic bioactive compounds (essential fatty acids and tocopherols), and the possibility of producing protein isolates. However, this study is a starting point and should be complemented with additional studies that demonstrate the possibility of obtaining lipophilic compounds and protein isolates from the solid residue, in addition to characterizing them and studying important aspects, such as their stability or possible toxicity.

## 5. Patents

The technological development and the product (encapsulated extract) are protected (ES 1 300 302 U) by a utility model (application number: U202232132/expedition date: 21 August 2023). The protection includes (i) the stages of the production process for obtaining and stabilizing (encapsulation) bioactive compounds from elderberry, (ii) the final product obtained (encapsulated elderberry extract), and (iii) its application in the meat industry. Therefore, the steps described in the M&M section are included in the invention protection but do not limit its scope.

## Figures and Tables

**Table 1:** Proximate composition (g/100 g), tocopherols (µg/g of oil), and fatty acids (g/100 g of oil) of the solid residue obtained after extraction (n = 16).

	Solid Residue		
	Values	Min	Max
Proximate Composition (g/100 g)			
Moisture	18.34 ± 6.10	10.31	23.93
Fat †	17.07 ± 1.25	15.28	18.75
Protein †	15.66 ± 0.55	14.78	16.43
Ash †	1.36 ± 0.10	1.23	1.51
Carbohydrates †	65.91 ± 1.25	64.42	68.32
Tocopherols (µg/g of oil)			
α-Tocopherol	317 ± 37.5	260.53	360.30
β-Tocopherol	6.24 ± 0.72	5.25	7.44
δ-Tocopherol	463 ± 48.2	396.36	518.96
γ-Tocopherol	4.61 ± 0.49	3.99	5.29
Total Tocopherols	791 ± 81.4	667.06	877.70
Fatty acids * (g/100 g of oil)			
C16:0 (palmitic acid)	6.12 ± 0.25	5.70	6.43
C18:0 (stearic acid)	1.56 ± 0.03	1.52	1.61
C18:1 *n*-9 (oleic acid)	10.72 ± 0.21	10.33	11.07
C18:1 *n*-7 (vaccenic acid)	0.68 ± 0.03	0.63	0.71
C18:2 *n*-6 (linoleic acid)	32.88 ± 0.43	32.04	33.46
C18:3 *n*-3 (α-linolenic acid)	32.56 ± 0.47	31.68	33.19
9*c*,11*t*-C18:2 (conjugated linoleic acid)	0.17 ± 0.02	0.13	0.21
C20:0 (arachidic acid)	0.17 ± 0.01	0.15	0.18
C20:1 *n*-9 (*cis*-11-eicosenoic acid)	0.13 ± 0.003	0.13	0.14
C22:0 (behenic acid)	0.15 ± 0.01	0.13	0.18
SFA	8.50 ± 0.32	7.92	8.85
MUFA	11.80 ± 0.27	11.30	12.20
PUFA	65.75 ± 0.91	63.98	66.98
Σ *n*-3	32.59 ± 0.47	31.72	33.23
Σ *n*-6	32.98 ± 0.43	32.14	33.57
*n*-6/*n*-3	1.01 ± 0.00	1.01	1.02
Total fatty acids	86.05 ± 1.45	83.21	88.02

† Results of the chemical composition are expressed as g/100 g of dry matter. * In the table, only the fatty acids that represent more than 0.1% are presented, but for the sum (SFA, MUFA, PUFA, Σ *n*-3, Σ *n*-6, and total fatty acids), total identified and quantified fatty acids were taken into account; SFA: saturated fatty acids; MUFA: monounsaturated fatty acids; PUFA: polyunsaturated fatty acids.

**Table 2:** Quantification of bioactive compounds and in vitro antioxidant capacity of the lyophilized (n = 16) and encapsulated (n = 16) elderberry extracts.

	Extracts		Sig.
	Lyophilized	Encapsulated	
Bioactive compounds			
Total phenolic compounds (mg GAE/100 g)	7486 ± 395	1132 ± 168	***
Anthocyanins (mg/100 g)	520 ± 39.2	60.0 ± 7.55	***
Antioxidant Capacity			
DPPH (mg Trolox/g)	39.92 ± 1.73	3.84 ± 0.47	***
ABTS (mg ascorbic acid/g)	145.9 ± 7.4	12.91 ± 1.66	***
FRAP (mmol Fe^2+^/100 g)	177.7 ± 8.76	12.27 ± 1.98	***
ORAC (mg Trolox/g)	286 ± 23.3	46.0 ± 13.1	***
IC_50_ (mg/mL)	4.24 ± 0.44	34.5 ± 7.81	***

*** Significance (*p* < 0.001).

**Table 3:** Identification and quantification (mg/100 g of extract) of the non-anthocyanin and anthocyanin phenolic compounds present in elderberry lyophilized extract (n = 16).

Non-Anthocyanin						
Peak No.	Rt (min)	λ Max (nm)	[M-H]^−^ (*m*/*z*)	MS^2^	Tentative Identification	Quantification (mg/100 g of Extract)
1	5.83	325	353	191(100), 179(9), 173(19), 161(5)	*cis* 5-*O*-Caffeoylquinic acid	218 ± 8.30
2	6.63	327	353	191(100), 179(6), 173(21), 161(9)	*trans* 5-*O*-Caffeoylquinic acid	286 ± 17.0
3	16.52	353	609	301(100)	Quercetin-3-*O*-rutinoside	218 ± 14.5
					Total non-anthocyanin	722 ± 31.5
**Anthocyanin**						
4	27.44	515	743	581(72), 449(100), 287(78)	Cyanidin-*O*-sambubioside-*O*-hexoside	525 ± 57.1
5	27.89	515	743	581(72), 449(100), 287(78)	Cyanidin-3-*O*-sambubioside-5-*O*-glucoside	943 ± 53.8
6	30.97	518	581	287(100)	Cyanidin-3-*O*-sambubioside	4827 ± 546
7	31.92	517	449	287(100)	Cyanidin-3-*O*-glucoside	4926 ± 756
					Total anthocyanin	11,221 ± 1143

Standard calibration curves used: chlorogenic acid (y = 168,823x − 161,172, R^2^ = 0.9999, LOD = 0.20 µg/mL; LOQ = 0.68 µg/mL; peaks 1 and 2), quercetin-3-*O*-glucoside (y = 34,843x − 160,173, R2 = 0.9998, LOD = 0.21 µg/mL; LOQ = 0.71 µg/mL, peak 3), and cyanidin-3-*O*-glucoside (y = 105,078x − 12,437; R2: 0.9993; LOD = 0.28 µg/mL; LOQ = 0.84 µg/mL, peaks 4, 5, 6 and 7). Results are expressed as mean ± SD.

## Data Availability

The data used to support the findings of this study can be made available by the corresponding author upon request.
